# Genetically engineered suicide gene in mesenchymal stem cells using a Tet-On system for anaplastic thyroid cancer

**DOI:** 10.1371/journal.pone.0181318

**Published:** 2017-07-20

**Authors:** Senthilkumar Kalimuthu, Ji Min Oh, Prakash Gangadaran, Liya Zhu, Ho Won Lee, Yong Hyun Jeon, Shin Young Jeong, Sang-Woo Lee, Jaetae Lee, Byeong-Cheol Ahn

**Affiliations:** Department of Nuclear Medicine, Kyungpook National University School of Medicine/Hospital, Daegu, Republic of Korea; Wayne State University, UNITED STATES

## Abstract

Anaplastic thyroid cancer (ATC) is the most aggressive malignancy of the thyroid, during which undifferentiated tumors arise from the thyroid follicular epithelium. ATC has a very poor prognosis due to its aggressive behavior and poor response to conventional therapies. Gene-directed enzyme/prodrug therapy using genetically engineered mesenchymal stromal cells (MSC) is a promising therapeutic strategy. The doxycycline (DOX)-controlled Tet inducible system is the most widely utilized regulatory system and could be a useful tool for therapeutic gene-based therapies. For example, use a synthetic “tetracycline-on” switch system to control the expression of the therapeutic gene thymidine kinase, which converts prodrugs to active drugs. The aim of this study was to develop therapeutic MSCs, harboring an inducible suicide gene, and to validate therapeutic gene expression using optical molecular imaging of ATC. We designed the Tet-On system using a retroviral vector expressing herpes simplex virus thymidine kinase (HSV1-sr39TK) with dual reporters (eGFP-Fluc2). Mouse bone marrow-derived mesenchymal stromal cells (BM-MSC) were transduced using this system with (MSC-Tet-TK/Fluc2) or without (MSC-TK/Fluc) the Tet-On system. Transduced cells were screened and characterized. Engineered MSCs were co-cultured with ATC (CAL62/Rluc) cells in the presence of the prodrug ganciclovir (GCV) and stimulated with DOX. The efficiency of cell killing monitored by assessing Rluc (CAL62/Rluc) and Fluc (MSC-Tet-TK/Fluc and MSC-TK/Fluc) activities using IVIS imaging. Fluc activity increased in MSC-Tet-TK/Fluc cells in a dose dependent manner following DOX treatment (R2 = 0.95), whereas no signal was observed in untreated cells. eGFP could also be visualized after induction with DOX, and the HSV1-TK protein could be detected by western blotting. In MSC-TK/Fluc cells, the Fluc activity increased with increasing cell number (R2 = 0.98), and eGFP could be visualized by fluorescence microscopy. The Fluc activity and cell viability of MSC-Tet-TK/Fluc and MSC-TK/Fluc cells decreased significantly following GCV treatment. A bystander effect of the therapeutic cells confirmed in co-cultures of CAL62 cells, an anaplastic thyroid cancer cell line, with either MSC-Tet-TK/Fluc cells or MSC-TK/Fluc cells. The Rluc activity in MSC-Tet-TK/Fluc co-cultures, derived from the CAL62/Rluc cells, decreased significantly with GCV treatment of DOX treated cultures, whereas no significant changes were observed in untreated cultures. In addition, the Fluc activity of MSC-Tet-TK/Fluc cells also decreased significantly with DOX treatment whereas no signal was present in untreated cultures. A bystander effect also be demonstrated in co-cultures with MSC-TK/Fluc cells and CAL62/Rluc; both the Rluc activity and the Fluc activity were significantly decreased following GCV treatment. We have successfully developed a Tet-On system of gene-directed enzyme/prodrug delivery using MSCs. We confirmed the therapeutic bystander effect in CAL62/Rluc cells with respect to MSC-Tet-TK/Fluc and MSC-TK/Fluc cells after GCV treatment with and without DOX. Our results confirm the therapeutic efficiency of a suicide gene, with or without the Tet-On system, for ATC therapy. In addition, our findings provide an innovative therapeutic approach for using the Tet-On system to eradicate tumors by simple, repeated administration of MSC-Tet-TK/Fluc cells with DOX and GCV.

## Introduction

The ultimate aim of gene therapy is to treat a disease by genetically modifying the cells [1]. Transgenes may be directly transferred into a range of target cells, including normal cells, cancer cells, or pluripotent stem cells. If a transgene introduced into a cancer cell, then it can lead to either cell death or restore normal cellular function, whereas introduction of the transgene into normal cells can protect the cell from drug-induced toxicities, or initiate an immune response. Gene or vector-based therapies for cancer include an extensive role for treatment modalities within the tumor microenvironment [2, 3].

Tetracycline-controlled transcriptional activation is a widely used method of inducible gene expression wherein transcription is reversibly turned on or off in the presence of the antibiotic tetracycline or one of its derivatives (e.g. doxycycline; DOX). The Tet technology consists of two complementary control systems, initially described as the tTA-dependent (Tet-Off System) and rtTA-dependent expression systems (Tet-On System). The tetracycline-inducible system is the most widely utilized regulatory system and might be a useful tool in the emerging era of synthetic biology [4, 5]. Suicide genes exhibit a cytotoxic effect after expression in the host cell. Certain suicide genes encode enzymes that convert nontoxic prodrugs into highly toxic metabolites, thereby precisely eliminating cells expressing these enzymes [6]. Suicide genes have been studied for the treatment of cancer through the introduction of these genes into cancer cells or neighboring cells. The expression of suicide genes in cancer cells results in the generation of cytotoxic products, killing the cancer cell; nearby cancer cells that do not express the suicide gene can also be killed by exposure to cytotoxic products by a process known as the bystander effect [7–12].

The first, and most commonly used, suicide gene is the herpes simplex virus-thymidine kinase (HSV1-TK), which converts the drug ganciclovir (GCV) into cytotoxic GCV-triphosphate [6, 13]. The combination of the suicide gene HSV1-TK with a tightly controlled system, such as Tet-On, which simply regulated by presence, or absence of doxycycline, could reduce the side effects associated with gene-based therapy.

Mesenchymal stem cells (MSCs) are multipotent, and are derived from a variety of tissues or organs, including adipose tissue, umbilical cord, placenta, and bone marrow [14]. MSCs are attractive vehicles for the delivery of therapeutic agents to cancer cells, based on their tumor-specific tropism, and have been used for suicide gene therapy [15–17]. MSCs engineered with apoptosis inducers (e.g. TRAIL), oncolytic viruses, interleukins (ILs), interferons (IFNs), or HSV1-TK are able to deliver these biological molecules to tumors and have demonstrated antitumor activity [7, 18–21]. Imaging technologies have been utilized for the diagnosis and monitoring of therapeutic effects and these tools are indispensable for developing new drugs before their clinical translation [22].

Globally, thyroid cancer incidence has been continuously increasing over the past few decades, and has become the fifth most common malignancy in females [23]. The most aggressive form of thyroid cancer, anaplastic thyroid cancer (ATC), is a highly aggressive form of the disease with poor prognosis [24]. Unfortunately, resistance to radio- and chemotherapy is a common feature; thus, the development of novel therapeutics is essential to improve the treatment of ATC patients. There have been no reports of ATC treatment with MSCs harboring a suicide gene. The aim of this study was to develop therapeutic MSCs expressing an inducible HSV1-TK suicide gene, and to validate therapeutic gene expression *in vitro* using optical molecular imaging and a model of ATC.

## Materials and methods

### Materials

Tetracycline-free fetal bovine serum (FBS), doxycycline, and pRetroX-TRE3G and pRetroX-Tet3G plasmids were purchased from Clontech (Mountain View, CA, USA). A CaPO_4_ transfection kit and gentamicin were purchased from Invitrogen (Carlsbad, CA, USA).

### Cell culture

Mouse bone marrow-derived MSCs (Invitrogen, USA) were cultured in DMEM-F12 (HyClone, Logan, UT, USA) supplemented with 10% FBS (HyClone), 1% gentamicin (GIBCO-BRL Life Technologies, Gaithersburg, MD, USA), and 1× Glutamax (Invitrogen). The human anaplastic thyroid cancer cell line CAL62 was purchased from (DSMZ-Germany), and mouse bone marrow derived mesenchymal stem cells (BM-MSCs) were purchased from Invitrogen. CAL62 cells were grown in DMEM supplemented with 10% FBS and 1% penicillin/streptomycin solution (HyClone).

### Plasmid constructs

Tet-responsive regulatory systems are normally comprised of two different expression cassettes, one containing the regulatory protein (tTA) and the second containing the Tet regulatory element fused to a minimal promoter, which regulates the expression of the reporter or therapeutic transgene. The inducible expression system was purchased from Clontech, and it contains a regulator plasmid (pRetroX-Tet3G) and a response plasmid (pRetroX-TRE3G). The therapeutic gene HSV1-sr39TK (HSV1-TK) and reporter gene, containing the full sequence of sr39TK-eGFP-IRES-Fluc2, were inserted into the response plasmid pRetroX-TRE3G after the TRE3G promoter. Based on previously described transcriptional regulators [25–27], the Tet-On 3G is a modified form of the Tet-On advanced transactivator protein that has been modified for markedly increased sensitivity to DOX [28]. The inducible promoter pTRE3G yields very low basal expression and maximal expression after induction [29]. It consists of seven repeats of a 19 bp tet operator sequence located upstream of a minimal CMV promoter to give higher performance for retroviruses. In the presence of DOX, Tet-On 3G binds specifically to pTRE3G and activates transcription of the downstream genes (sr39TK). pTRE3GV lacks binding sites for endogenous mammalian transcription factors, so it is virtually silent in the absence of induction.

### Viral transduction

CAL62 cells were transduced with lentiviral particles expressing mCherry-Rluc under the control of the CMV promoter (GeneCopoeia, Rockville, MD, USA) and then selected with 2 μg/mL of puromycin (CAL62/Rluc cells) for 2–3 weeks. MSC cells were transduced with retrovirus isolated from Gryphon E cells (Allele Biotechnology, San Diego, CA, USA). The plasmids Retro-Tet3G and Retro-HSV1-sr39TK-eGFP-IRES-Fluc2 were separately transfected by the CaPO_4_ method into Gryphon E cells. After overnight transfection, the medium was changed, and the virus was collected daily for 2 days, pooled, passed through a 0.45-μm filter, and concentrated using an Amicon filter. The concentrated virus was used to transduce MSCs with a 1:1 dilution of Retro- Tet3G and Retro-HSV1-sr39TK-eGFP-IRES-Fluc2 for making Tet-On MSC-Tet-TK/Fluc2 cells; stable transfectants without Tet-On (MSC-TK/Fluc) were also developed. The MSC-Tet-TK-Fluc cells were sorted after treatment with DOX at 2 μg/mL, and MSC-TK/Fluc cells were sorted by flow cytometry based on GFP expression. After one round of sorting, GFP was expressed in more than 50% of cells, and these cells were used for further study. The sorted cells were screened and characterized.

### *Renilla* luciferase (Rluc) and *firefly* luciferase (Fluc) activity in transduced cells

To confirm Rluc activity, CAL62 and CAL62/Rluc cells at different densities (1.25×10^4^, 2.5×10^4^, 5×10^4^, 1×10^5^, and 2×10^5^ cells/well) were plated in a clear-bottom 24-well plate in DMEM. After 24 h, the substrate coelenterazine (CTZ) (Perkin Elmer, Waltham, MA, USA) was added to each well, and Rluc activity was determined by BLI (Bioluminescence imaging), using the IVIS Lumina II system (Caliper Life Sciences, Hopkinton, MA, USA). To confirm the Fluc activity of MSC-TK/Fluc, cells at different densities (0.625×10^4^, 1.25×10^4^, 2.5×10^4^, 5×10^4^, 1×10^5^, and 2×10^5^ cells/well) were plated in a clear-bottom 24-well plate and cultured in DMEM medium for 24 h, and then Fluc activity was measured. For MSC-Tet-TK/Fluc cells, we plated 2×10^4^ cells/well in a 48 well plate. After 24 h, 0.625 to 2 μg/mL of DOX was added to each well, and after further incubated for 24 h. Fluc activity was then determined by BLI using the IVIS Lumina II system. After BLI imaging we carefully drew a separate region of interest (ROI) in each well and the signal was quantified using living image software.

### Dose- and time-dependent Fluc activity in MSC-Tet-TK/Fluc cells

Initially the Fluc activity of MSC-Tet-TK/Fluc cells was assessed with 0.625 to 2 μg/ mL of DOX. We further analyzed the dose- and time-dependent Fluc activity. MSC-Tet-TK/Fluc cells were plated in a 48-well plate (1×10^4^ cells/well) and cultured overnight; the cells were incubated with increasing concentrations of DOX (0.5 to 16 μg/ mL) for 24, 48, or 72 h. In addition, after 24 h of DOX induction, the medium was removed and fresh medium was added for another 24 or 48 h to assess residual Fluc activity. Fluc activity was measured by BLI using the IVIS Lumina II system.

### Western blot analysis

For western blot analysis, 50-μg protein samples were separated by 10% SDS-PAGE and transferred to PVDF membranes (Millipore, Billerica, MA, USA). The blots were incubated with primary antibodies against HSV1-TK (Santa Cruz Biotechnology, Inc., Santa Cruz, CA, USA), followed by incubation with HRP-tagged secondary antibodies. The protein-antibody complexes were visualized using an enhanced chemiluminescence kit (Pierce, Rockford, IL, USA).

### ^3^H-penciclovir (PCV) uptake assay

For functional assessment of the transfected cells, we performed a ^3^H-Penciclovir uptake assays as described in Sekar et al., 2012 [30]. Briefly, MSC-TK/Fluc and MSC-Tet-TK/Fluc cells stably expressing HSV1-TK, along with control cells were plated (2×10^5^ cells/well) in 6-well plates. Twenty-four hours after plating, cells were induced with or without DOX for gene activation and then treated with 1 μCi of ^3^H-penciclovir (Moravek Biochemicals, La Brea, CA, USA). After 1, 2, and 4 h of incubation at 37°C with 5% CO_2_, cells were washed twice with ice-cold PBS, lysed in 0.5 mL of 0.1% SDS and analyzed by a scintillation counter after adding 10 mL of scintillation fluid. The obtained counts per minute (CPM) were analyzed based on the CPM of cell lysates/CPM of medium/μg of protein.

### Cell viability and Fluc activity in MSC-Tet-TK/Fluc and MSC-TK/Fluc cells after GCV treatment

MSC-TK/Fluc and MSC-Tet-TK/Fluc cells were treated with or without DOX followed by treatment with different concentrations of GCV (0.25, 0.5, 1, 2, 4, and 8 μM) for 48 h to assess the therapeutic efficiency of the HSV-1-TK suicide gene. For this, we plated MSC-TK/Fluc or MSC-Tet-TK/Fluc cells in a 48-well plate at a density of 1×10^4^ cells/well. After overnight culture, the MSC-Tet-TK/Fluc cells were induced with 2 μg DOX to activate expression of the therapeutic gene. After 24 h stimulation with DOX, different concentrations of GCV were administered to all of the wells. After 48 h, Fluc activity was measured by BLI using the IVIS Lumina II system.

To determine the effects of GCV on cell viability, MSC-TK/Fluc or MSC-Tet-TK/Fluc cells were seeded in 96-well plates (5×10^3^/well) and treated with different doses of GCV after 24 h induction with (or without) DOX (2 μg/ml). Cell viability was assessed 48 h post-GCV treatment using Cell Counting Kit-8 (CCK-8) (Dojindo, Kumamoto, Japan).

### Bystander effect

To assess the therapeutic efficiency of GCV we first co-cultured naïve MSCs with CAL62/Rluc cells and treated them with GCV (0.25 to 8 μM) for 48 h. Next, we assessed the bystander effect. MSC-TK/Fluc and MSC-Tet-TK/Fluc cells were separately mixed with CAL62/Rluc (mCherry-Rluc) cells at a ratio of 1:1 in a 48-well plate and treated with various concentrations of GCV after induction, with or without DOX, for 24 h. Subsequently, cells were treated with GCV for 48 h. The survival of CAL62/Rluc and MSC-Tet-TK/Fluc cells was evaluated based on Rluc and Fluc activity using the IVIS Lumina II system. Further, to confirm bystander-mediated cytotoxicity of GCV treatment, we performed an analysis of morphological changes using fluorescence microscopy.

### *In vivo* therapeutic effect of MSC-Tet-TK/Fluc and MSC-TK/Fluc

In order to confirm the *in vivo* therapeutic effect we performed the *in vivo* experiment for MSC-Tet-TK and MSC-TK cells. This animal experimental procedure was reviewed and approved by the Kyungpook National University (KNU-2012-43) Animal Care and Use Committee and performed in accordance with the Guiding Principles for the Care and Use of Laboratory Animals. For IVIS imaging, we used a controlled amount of isoflurane anesthesia and oxygen system to the imaging chamber for animal imaging. For *in vivo* experiment, we separated MSC-Tet-TK and MSC-TK group. For MSC-Tet-TK group, CAL62/Rluc cells with MSC (left) and DOX induced MSC-Tet-TK/Fluc (right) cells was separately co-injected at 1:1 (1.5×10^6^ cells each) ratio in left and right flank of the nude mice. In addition, the same strategy applied for MSC-TK group. Then, Rluc activity was measured by IVIS imaging by using coelentrazine as a substrate. MSC-Tet-TK group mice were received DOX (2 mg/kg b.wt) and GCV (30mg/kg b.wt) by intra peritoneal injection and MSC-TK group mice were received GCV (30mg/kg b.wt) for four days. After four days, treatment we analyzed the Rluc activity and quantified.

### Statistical analysis

Individual experiments were performed three times and data is presented as means ± standard deviation (SD). A P value <0.05 was considered statistically significant by Student t-test.

## Results

### Characterization of MSC-Tet-TK/Fluc and MSC-TK/Fluc cells

**Fig 1:**
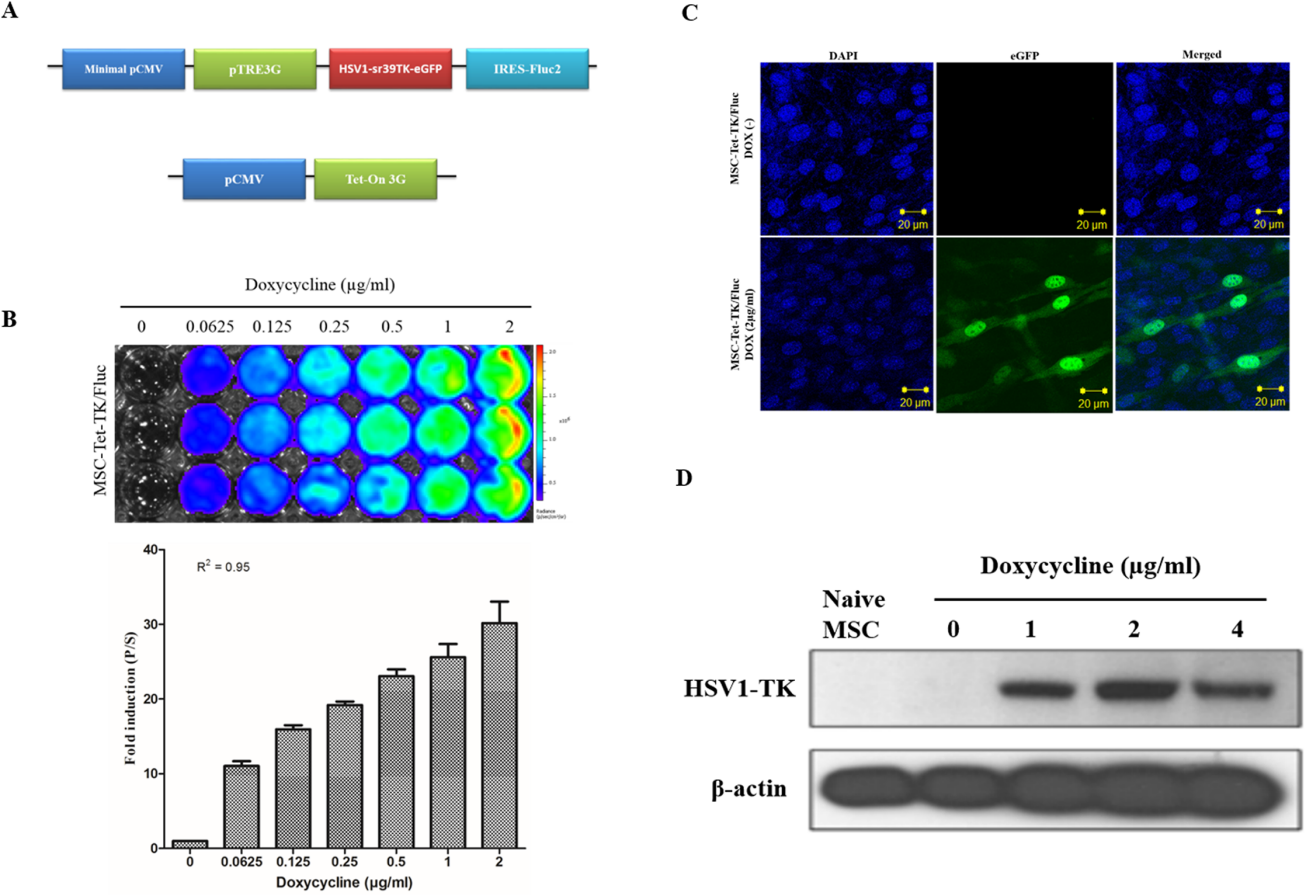
Transduction of MSCs, with triple fusion (TF) reporter genes. (A) Schematic of the TF reporter gene construct pTRE3G-sr39tk-eGFP-Fluc and the plasmid expressing the Tet-On 3G activator. (B) Confocal microscopy image of eGFP in MSC-Tet-TK/Fluc cells after induction with DOX (2 μg/mL) for 24 h. (C) Fluc activity in stably transduced MSCs (MSC-Tet-TK/Fluc) after DOX treatment for 24 h and detected using BLI quantitative imaging of Fluc activity. (D) Western Blot analysis of HSV1-TK protein after 24 h induction of MSC-Tet-TK/Fluc cells with DOX (0,1,2 and 4 μg/ml).

**Fig 2:**
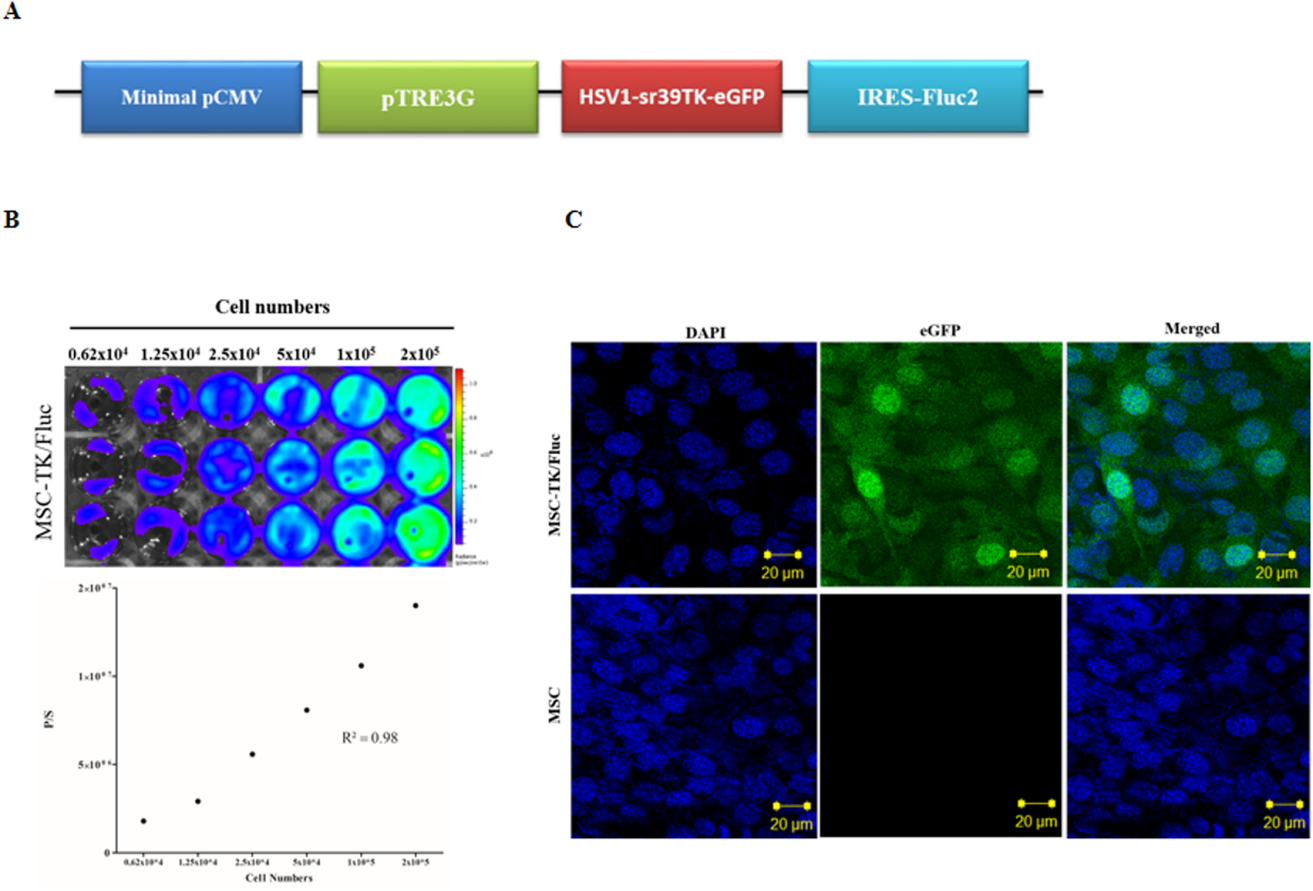
Transduction of MSCs, with triple fusion (TF) reporter genes without the Tet-On system. (A) Scheme for the TF reporter gene plasmid pCMV-pTRE3G-sr39tk-eGFP-Fluc, which has a minimal promoter. (B) Fluc activity detected by BLI imaging and quantitative analysis of Fluc signal as a function of MSC-TK/Fluc cell number. (C) eGFP expression in MSC-TK/Fluc shown by confocal microscopy.

The BLI signal in MSC-Tet-TK/Fluc cells was low in untreated cells but increased with increasing concentrations of DOX (Fig 1B, R^2^ = 0.95). Fluc signal was normalized with DOX(-) Fluc activity which were increased with increased 11, 15.9, 19.1, 23, 25.6 and 30 fold after DOX induction over a concentration range of 0.0625–2 μg/mL, respectively. Based on the maximal induction being obtained at 2 μg/mL DOX, this concentration was selected for activation of the Tet-On system for the remainder of the experiments in this study. The presence of the HSV1-TK protein was confirmed by western blot analysis after DOX induction (2 μg/mL) for 24 h; notably, the HSV1-TK protein was not detected in the untreated cells or naïve MSC cells (Fig 1C). The Fluc activity in MSC-TK/Fluc cells increased with increasing cell number and showed a good correlation with cell number (Fig 2B, R^2^ = 0.98). MSC-Tet-TK/Fluc and MSC-TK/Fluc cells, eGFP expression was visualized by confocal microscopy (Figs 1C and 2C).

### Characterization of CAL62/Rluc cells

The Rluc activity in stably transfected CAL62/Rluc cells was measured using BLI. Rluc activity in CAL62/Rluc cells increased with increasing cell number compared to CAL62 cells alone (S1B Fig, R^2^ = 0.96). The expression of mCherry was also visualized by fluorescence microscopy (S1C Fig). The presence of the Rluc gene was confirmed by RT-PCR analysis (S1D Fig) while the presence of the Rluc protein was confirmed by western blot analysis in CAL62/Rluc cells; no band was observed with parental CAL62 cells (S1E Fig).

### Time- and dose-dependent induction of Fluc during DOX treatment and following DOX withdrawal in MSC-Tet-TK/Fluc cells

We assessed the time and dose-dependent effect of DOX induction on Fluc activity in MSC-Tet-TK/Fluc cells as well as the time course of Fluc activity decay following DOX withdrawal. At 2 μg/mL DOX induction Fluc increased 26-, 20-, and 18-fold after 24, 48, and 72 h of continuous culture (S2A Fig), respectively. Following DOX withdrawal after 24 h of treatment, the fold change was 26% in 24 h later and 16% at 48 h later in 2μg/ml DOX (S2B Fig). Overall, DOX increased Flux activity in a dose-dependent manner at 24, 48, and 72 h of culture. Maximum Fluc activity was observed with DOX concentrations of 2 and 4 μg/mL after 24 and 48 h of culture, respectively. Based on these observations, we selected a concentration of 2 μg/mL of DOX for further *in vitro* experiments.

### Functional characterization of MSC-Tet-TK/Fluc using a ^3^H-PCV uptake assay

**Fig 3:**
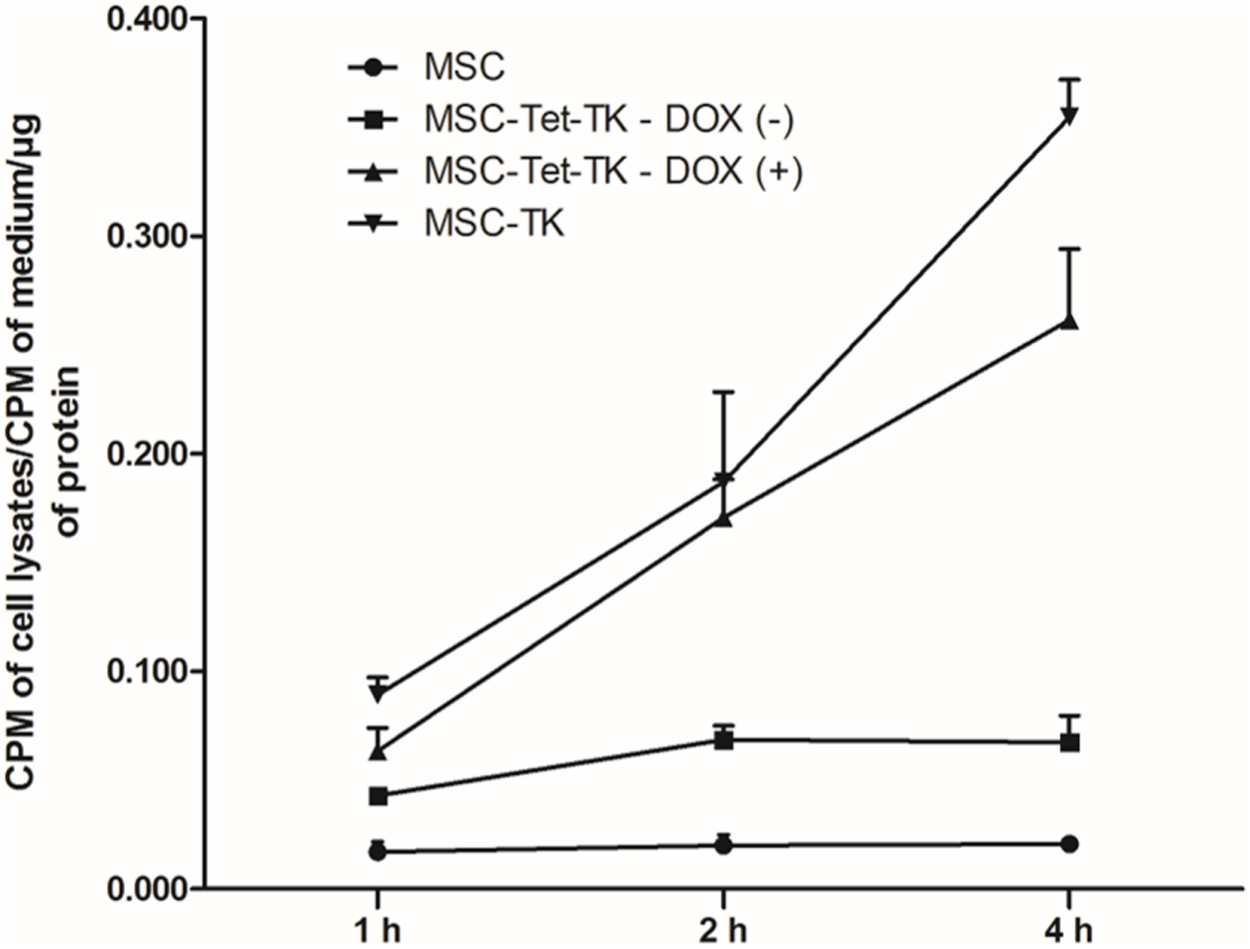
^3^H Penciclovir uptake assay in MSCs, MSC-Tet-TK/Fluc, and MSC-TK/Fluc cells. The penciclovir uptake of naïve MSCs, MSC-TK/Fluc, untreated and DOX-treated (2 μg/mL) MSC-Tet-TK/Fluc cells over 1, 2, and 4 h time periods.

A ^3^H-penciclovir (PCV) uptake assay was performed to measure the functional efficiency of MSC-TK/Fluc and MSC-Tet-TK/Fluc cells. HSV1-TK converts PCV into PCV-monophosphate; subsequently endogenous cellular TK phosphorylates the monophosphate form to generate PCV-triphosphate. The accumulation of ^3^H-PCV therefore indirectly indicates the functional efficiency of the HSV1-TK enzyme expressed in cells. MSC-TK/Fluc and MSC-Tet-TK/Fluc stable cells incubated with DOX (2 μg/mL) had higher ^3^H-PCV uptake compared to either control MSCs or untreated MSC-Tet-TK/Fluc cells (Fig 3).

### Effect of GCV and DOX on MSC cell viability using CCK-8

**Fig 4:**
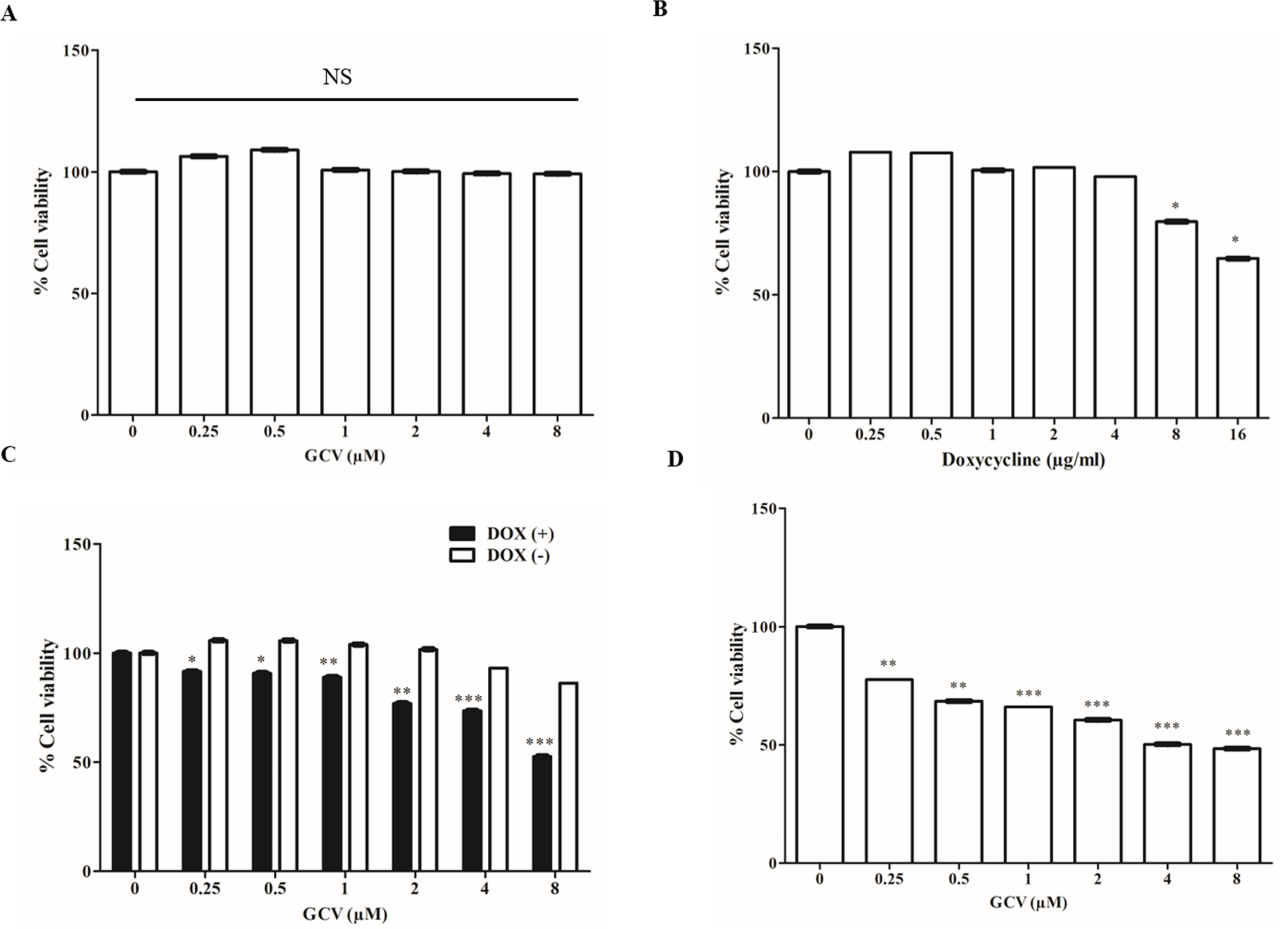
Cell viability of MSC, MSC-Tet-TK/Fluc, and MSC-TK/Fluc cells using a CCK-8 assay. (A) Cell viability of naïve MSCs treated with GCV for 48 h. (B) Cell viability of MSCs treated with DOX for 48 h. (C) Cell viability of MSC-Tet-TK/Fluc cells treated after GCV treatment for 48 h with and without DOX. (D) Cell viability of MSC-TK/Fluc cells after GCV treatment for 48 h. The values are expressed as the mean ± standard deviation (SD), of three experiments. NS: denotes no significant change. * indicates significance at p<0.05 (by Student's t test).

We found that GCV (8 μM) did not affect cell viability in naïve MSCs after 48 h of treatment (Fig 4A), Since, we used DOX for gene activation, we also wanted to examine the effect of DOX on cell viability in these same cells; using increasing concentrations of DOX for 48 h we observed that cell viability was not decreased at 4 μg/mL DOX, but a significant reduction in cell viability was observed at both 8 and 16 μg/mL DOX (p < 0.05) (Fig 4B).

### Viability of MSC-Tet-TK/Fluc and MSC-TK/Fluc cells

**Fig 5:**
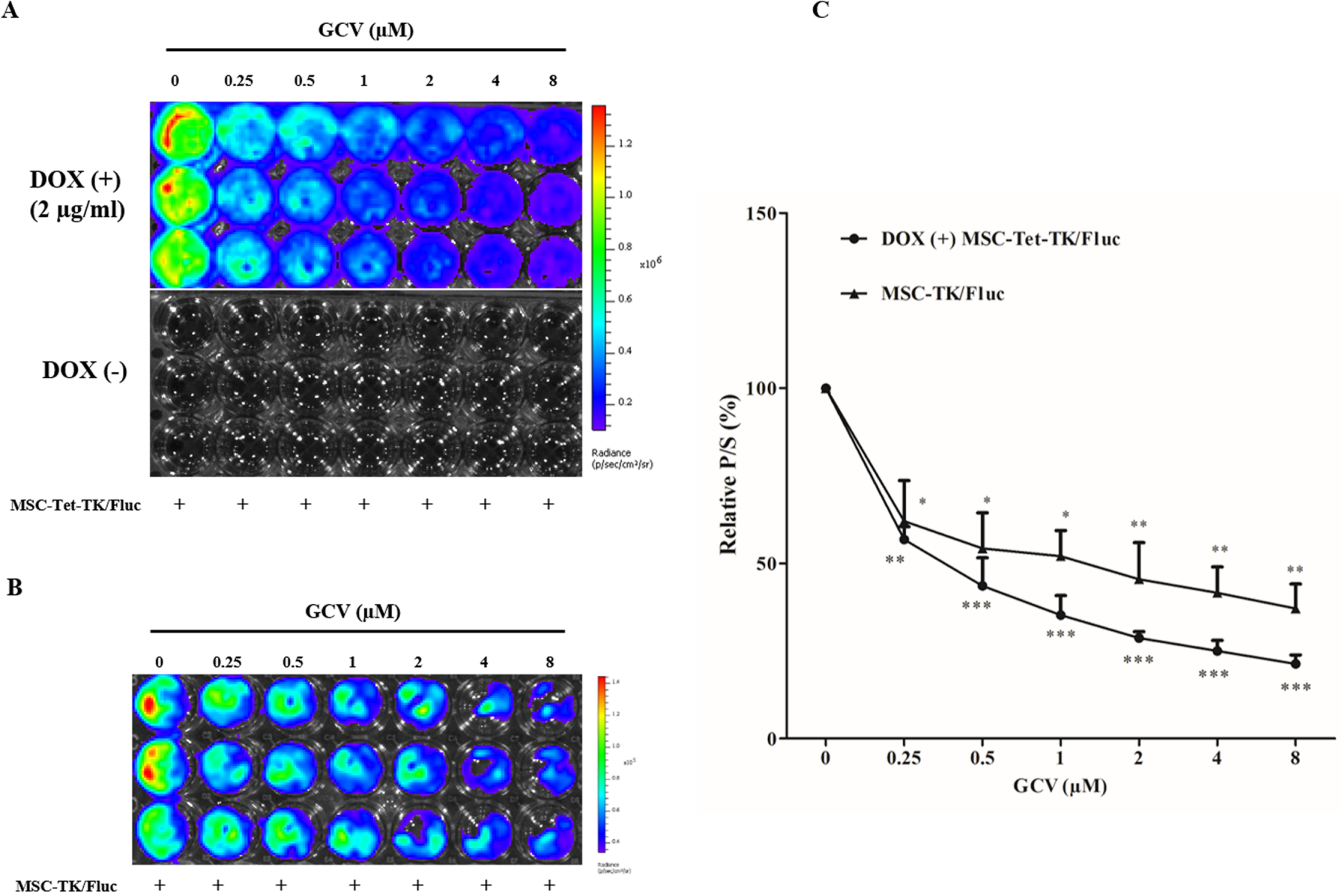
Fluc activity of MSC-Tet-TK/Fluc and MSC-TK/Fluc cells. (A). Fluc activity of MSC-Tet-TK/Fluc cells treated with GCV for 48 h after induction with and without DOX. (B) Fluc activity of MSC-TK/Fluc cells treated with GCV for 48 h. (C) Quantitation of the Fluc2 activity of MSC-Tet-TK/Fluc and MSC-TK/Fluc cells. The values are expressed as the mean ± standard deviation (SD), of three individual experiments and measurements. * indicates significance level at *p<0.05, **p<0.01., ***p<0.001 (by Student's t test).

Fluc activity in MSC-Tet-TK/Fluc cells was dropped 57% of control and 21% of control, at 0.25 μM and 8 μM GCV (Fig 5A) (p < 0.001) respectively. In contrast, DOX-untreated cells did not exhibit any basal Fluc activity (Fig 5A). In parallel, Fluc activity, and hence cell viability, in MSC-TK/Fluc cells decreased significantly being 62% of control and 37% of control, respectively, at 0.25 μM and 8 μM GCV (Fig 5B). Comparing the two cell types, the decreases in Fluc activity were greater in the MSC-Tet-TK/Fluc DOX inducible cells than in the MSC cells stably expressing HSV1-TK (MSC-TK/Fluc). These changes in cell viability were confirmed using a CCK assay 48 h after GCV treatment. GCV (0.25 to 8 μM) treatment reduced the cell viability in DOX-treated MSC-Tet-TK/Fluc cells, but not significantly in untreated MSC-Tet-TK/Fluc cells (Fig 4C). MSC-TK/Fluc cell viability also decreased gradually with increasing concentrations of GCV (0.25 to 8 μM) (Fig 4D). Taken together these data confirm the efficacy of GCV in HSV1-TK-expressing cells.

### Bystander effects of the Tet-On MSC-Tet-TK and MSC-TK/Fluc cell suicide system

**Fig 6:**
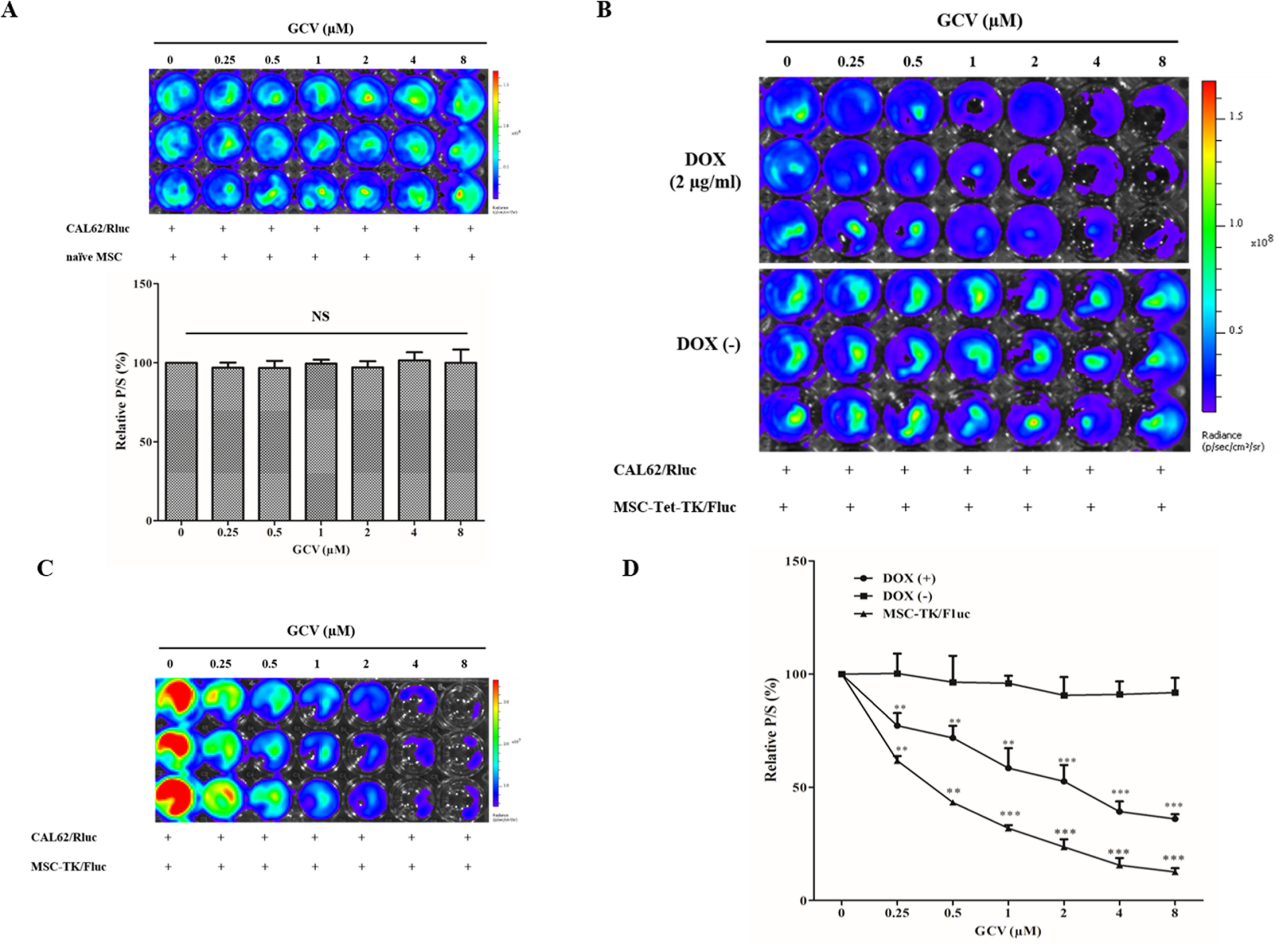
Bystander effect of suicide gene transduced cells. (A) Rluc activity of CAL62/Rluc cells co-cultured with naïve MSCs (1:1) followed by GCV treatment (0.25 to 8μM) for 48 h. (B) Rluc activity of CAL62/Rluc cells co-cultured (1:1) with MSC-Tet-TK/Fluc cells followed by GCV treatment for 48 h, with and without DOX (2 μg/mL) treatment (C) Rluc activity of CAL62/Rluc cells co-cultured (1:1) with MSC-TK/Fluc cells after GCV treatment for 48 h. The values are expressed as the means ± standard deviation (SD), of three individual experiments and measurements. * indicates significance level at *p<0.05, **p<0.01., ***p<0.001 (by Student's t test).

We assessed the effect of GCV on CAL62/Rluc cell viability through co-culture with naïve MSCs for 48 h, and found no significant reduction in Rluc activity (Fig 6A). Thus, we confirmed that GCV alone had no effect on Rluc activity in CAL62/Rluc cells when co-cultured with naïve MSCs. In DOX-treated co-cultures of MSC-Tet-TK/Fluc cells and CAL62/Rluc cells, the relative Rluc activity of CAL62/Rluc cells decreased steadily, being 77.2, 71.8, 58.5, 52.6, 39.2 and 36% of control (p < 0.01, 0.001) respectively at the GCV concentrations of 0.25 μM to 8 μM (Fig 6B and 6D). In contrast, in untreated co-cultures of MSC-Tet-TK/Fluc cells and CAL62/Rluc cells, the Rluc activity did not change significantly, being 100% of control and 92% of control respectively at GCV concentrations of 0.25 μM and 8 μM respectively. In co-cultures of MSC-TK/Fluc cells and CAL62/Rluc cells, the Rluc activity decreased, being 62, 43, 32, 24, 16 and 13% of control (p < 0.01, 0.001) respectively at 0.25 μM to8 μM GCV respectively (Fig 6C and 6D).

**Fig 7:**
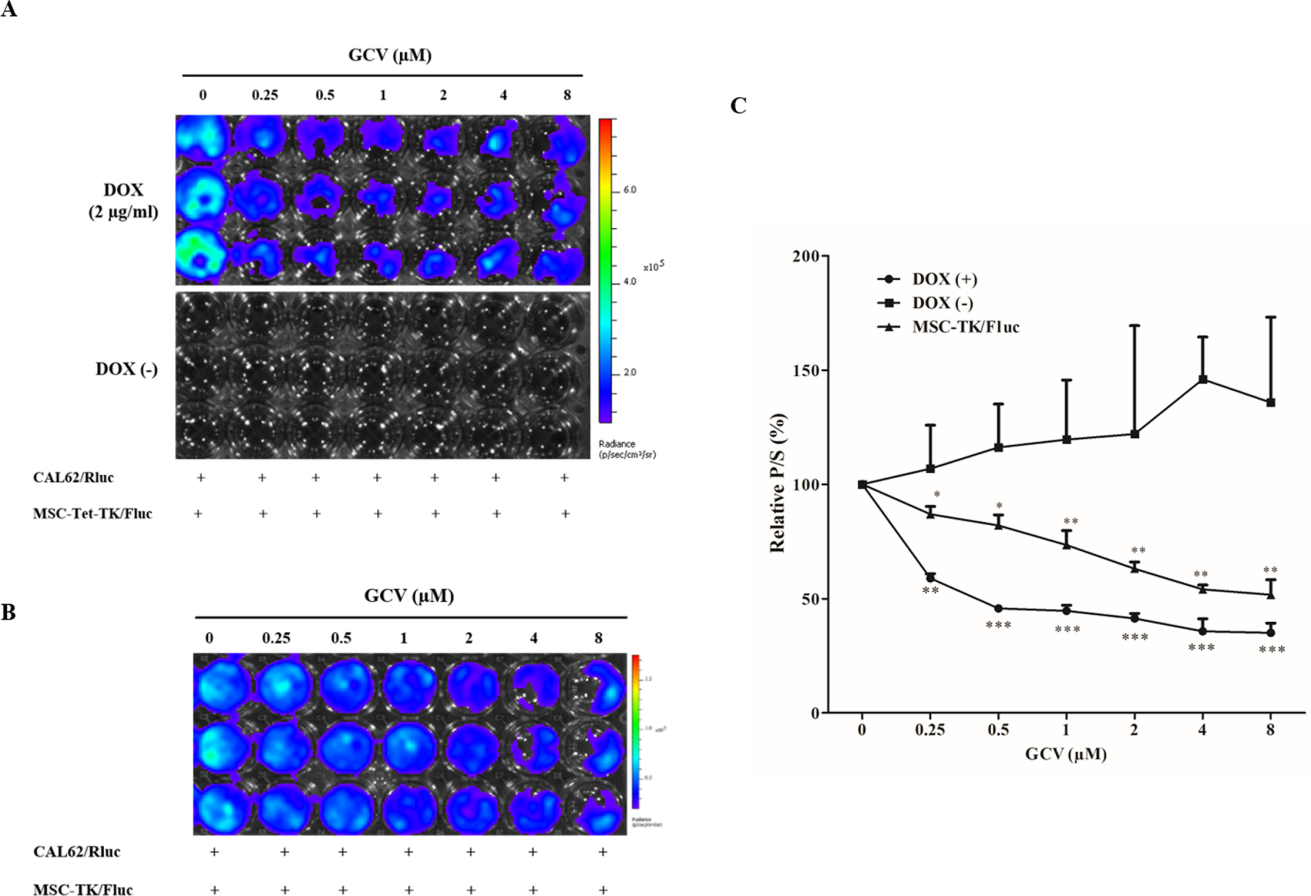
Fluc activity of MSC-Tet-TK/Fluc and MSC-TK/Fluc cells. (A) Fluc2 activity of MSC-Tet-TK/Fluc cells co-cultured (1:1) with CAL62/Rluc cells and treated with GCV for 48 h. (B) Fluc2 activity of MSC-TK/Fluc cells co-cultured (1:1) with CAL62/Rluc cells and treated with GCV for 48 h. The values are expressed as the mean ± standard deviation (SD), of three individual experiments and measurements. * indicates significance level at *p<0.05, **p<0.01., ***p<0.001 (by Student's t test).

In parallel, we also measured Fluc activity derived from the MSC-Tet-TK/Fluc cells in these co-cultures. In DOX treated co-cultures, Fluc activity was 59% of control and 35% of control at 0.25 μM and 8 μM GCV, respectively, demonstrating the expected activity of the Tet-On HSV1-TK/GCV suicide system (Fig 7A) in these DOX treated cells; basal Fluc activity was observed in the untreated co-cultures. Similarly, we also measured Fluc activity derived from the MSC-TK/Fluc cells in their co-culture. Fluc activity was 87% of control and 51% of control at 0.25 μM and 8 μM GCV, respectively (p < 0.05, 0.01) (Fig 7B).

Furthermore, we also confirmed the bystander effect by examining morphological changes. The bystander effect was evident in co-cultures using both MSC-Tet-TK/Fluc and MSC-TK/Fluc cells following GCV treatment. Although some cells remained attached to the plate, many of them had altered morphology with evidence of cell shrinkage and many cells had the appearance of thin and elongated cytoplasm, both of which are morphological characteristics of non-viable cells (S3B and S3C Fig). From these results, we confirmed that the therapeutic suicidal gene, either with or without the Tet-On system, in engineered MSC cells, has a significant bystander effect.

### MSC-Tet-TK and MSC-TK decrease *in vivo* Rluc activity of anaplastic thyroid cancer cell

**Fig 8:**
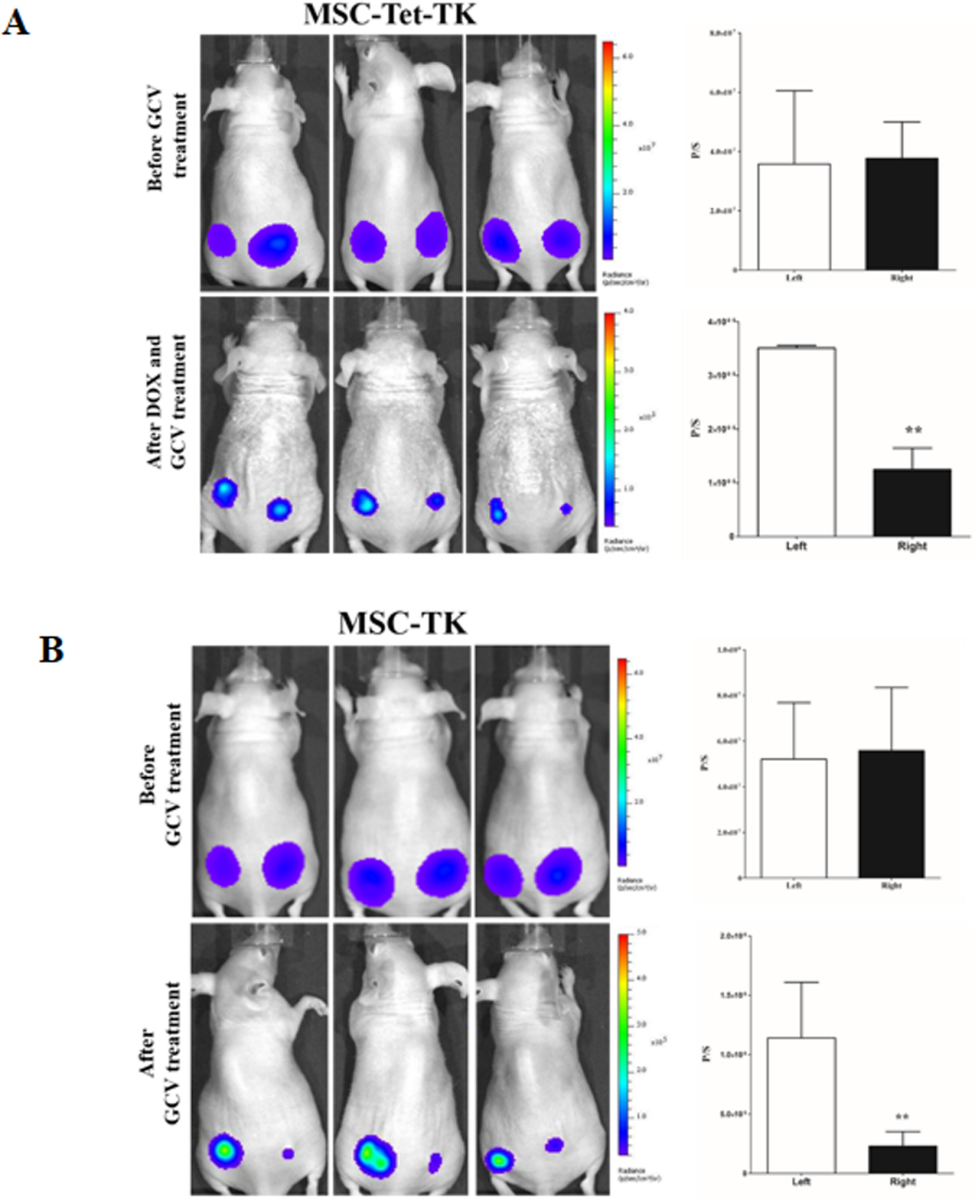
Rluc activity of CAL62/Rluc cells after therapeutic genes co-injected with CAL62/Rluc cells with and without DOX and GCV for four days treatment. (A) BLI of Rluc activity and quantitative Rluc activity of MSC-Tet-TK group. CAL62/Rluc cells co-injected with MSC (Left) and MSC-Tet-TK (Right). (B) BLI of Rluc activity and quantitative Rluc activity of MSC-TK group. CAL62/Rluc cells co-injected with MSC (Left) and MSC-TK (Right) side of the mice. Three mice data's were presented as means ± standard deviation (SD). The P value <0.05 was considered statistically significant by Student t-test.

To confirm the therapeutic efficiency of MSC-Tet-TK and MSC-TK cells in *in vivo* model, we performed the modified *in vivo* experiment. We found that GCV treatment significantly (*p*<0.05) decreased the Rluc activity of anaplastic thyroid cancer cells (CAL62/Rluc) in MSC-Tet-TK and MSC-TK co-injected site (right) compared than MSC co-injected (left) site (Fig 8A and 8B). Therefore, suicide gene based therapy may be useful for anaplastic thyroid cancer.

## Discussion

The efficacy of MSCs from various sources, that have been engineered with genes appropriate for an enzyme-prodrug approach for cancer therapy, has been reported in several preclinical studies [12, 31, 32]. In the current study, we developed MSCs with a tetracycline inducible therapeutic moiety (HSV1-TK) along with a reporter system that could be monitored using bioluminescent imaging. In addition, the therapeutic effect of MSCs on ATC was also confirmed.

In order to develop successful cell-based cancer therapies, non-invasive imaging methods to monitor both therapeutic cells and target cells are essential. For example, molecular imaging has been successfully used for monitoring of administered cells and tumors in living animals [7, 22, 33]. In this study, we generated MSCs harboring the therapeutic gene HSV1-TK, and the expression of this gene was tightly linked to the expression of reporter genes, eGFP and Fluc. Therefore, the expression of HSV1-TK could be monitored by measuring reporter expression. We generated MSCs that expressed HSV1-TK under the control of the Tet-On system (Fig 1) as well as MSCs that stably expressed HSV1-TK (Fig 2). The expression of HSV1-TK was successfully monitored in both cell types based on Fluc activity after administration of D-luciferin. Furthermore, an anaplastic thyroid cancer cell line, CAL62, (S1 Fig) was successfully transduced with a double-fusion reporter gene (mCherry-Rluc); therefore, tumor progression could potentially be visualized with BLI using coelenterazine as a luciferase substrate [7, 34]. In our study, the dual luciferase approach for imaging was successfully established for simultaneous monitoring of therapeutic gene expression in MSC-Tet-TK/Fluc cells and to assess the therapeutic effects of MSCs in conjunction with GCV treatment (Fig 6B).

Both infinite proliferation and decreased cell death are characteristics of cancer cells. ATC demonstrates local invasion and metastasis to the lung, lymph nodes, and other tissues, and therefore, the mortality rate is very high [35]. In this study, we reported that the Tet-On suicide gene system in MSCs successfully exhibited a bystander effect after induction with DOX and with GCV treatment, which could potentially be used for the treatment of thyroid cancer. After GCV treatment (8 μM, 48 h), the Rluc bioluminescent signal dropped to 36% and 13% of control (p < 0.01, 0.001 respectively) in co-cultures of either MSC-Tet-TK/Fluc or MSC-TK/Fluc cells with the thyroid cancer cell line CAL62 expressing Rluc. This suggests that this MSC-based suicide gene therapy tool inhibits thyroid cancer cell growth through a bystander mechanism, using either MSC-Tet-TK/Fluc cells (Fig 6B) or MSC-TK/Fluc cells (Fig 6C) as the source of the toxin. We also confirmed the therapeutic efficiency of MSC-Tet-TK and MSC-TK in an *in vivo* model (Fig 8A and 8B).

Antitumor drugs are able to kill some types of cancer cells; however, antitumor drugs generally produce significant toxicity in normal organs, generate host morbidity, and induce treatment related mortality [36–38]. Suicide gene therapy also generates systemic toxicity and byproducts. Therefore, controlling suicide gene expression might be a valuable tool to prevent side effects. Manipulating the expression of a suicide gene using an inducible system can block the generation of the suicide protein before its arrival at the target tissue (for example a tumor); therefore, suicide gene therapy utilizing an inducible system represent a safe choice among the diverse treatment options for cancer treatment. The Tet-On system uses an *E*. *coli* gene regulatory system [39], and has several advantages for clinical applications [39, 40]; in addition, gene expression can be easily regulated within the system. DOX induces gene expression in a dose-dependent manner through the activation of the Tet-response element in cells harboring the Tet-On system. Thus, the expression of a gene could be turned on after the arrival of therapeutic cells to the cancer site, potentially avoiding systemic toxicity of the therapeutic agent. The results from the present study suggest that the Tet-On/HSV1-TK/GCV system in MSCs induces a bystander effect and is a feasible method for the treatment of thyroid cancer.

Our data also show that there is an obvious dose-dependent relationship between HSV1-TK gene activity and DOX concentration in MSC-Tet-TK/Fluc cells and furthermore (Fig 1B) gene expression decreases upon withdrawal of DOX for either 24 h or 48 h (S2 Fig); in this study, we selected the DOX concentration with maximum induction ability namely 2 μg/mL. These results indicate that HSV1-TK expression might selectively enhance the sensitivity of ATC cells to GCV *in vitro*, in agreement with other reports suggesting that the Tet-On system exhibits high activity after induction, but low basal transcriptional activity [41]. The benefits of MSCs for cancer treatment are controversial as MSCs can also play a positive role in cancer progression depending on the cellular context [42, 43]. MSC cells expressing the HSV1-TK gene after DOX induction should theoretically be eradicated by administration of GCV; therefore, MSC-related adverse effects could be avoided. Thus, our system might be a safe alternative strategy for the treatment of human ATC.

Genetically engineered MSCs expressing HSV1-TK have the ability to inhibit the growth of cancer cells in both *in vitro* and *in vivo* tumor models [7, 44–46]. With our engineered MSCs, the expression of HSV1-TK can be easily monitored by reporter genes and controlled by DOX, and furthermore these engineered MSCs induce a bystander effect in ATC cells. The advantages of the Tet-On reporter system will be helpful for developing MSC-based therapeutic gene expression systems that could reduce the adverse effects of gene-based therapies. The limitation of this system is that the *in vitr*o effects seen here might not be well mimicked *in vivo*. In the cell culture medium, DOX directly affects every cell and therefore efficiently up regulates transgene expression. However, in the *in vivo* situation responses response may not be so reproducible and robust. There may be inherent animal-to-animal variation; the DOX concentration may also vary owing to pharmacokinetic differences, such as biological half-life, across individuals, and the factors affecting tumor penetration by DOX may be complex and perhaps vary from tumor to tumor. Another limitation is that usually DOX is given through water, and mice have to drink sufficient amount of water for optimal transgene expression. This may introduce another source of complication. Immunogenicity against the transactivator protein of the tet-on inducible system has been observed after adminstration of tet-on encoding vectors and it raises concerns about the clinical value of the system [47, 48]. The immune responses against tet regulatory elements can evade immune system by modifying the activator fused with Gly-Ala repeat of the Epstein–Barr virus Nuclear Antigen-1 protein [49]. Tet-off system also can be applied as a tetracycline inducible system to escape from an intact immune system [48].

## Conclusion

In this study, we successfully generated MSCs using the Tet-On HSV1-TK/Fluc system. Non-invasive monitoring of HSV1-TK expression in the cells and the cytotoxic effect of the cells on an anaplastic thyroid cancer cell line were successfully demonstrated. The novel platform of dual optical molecular imaging, visualizing both therapeutic gene expression in therapeutic cells and the cytotoxic effect on target cancer cells, might be useful for developing engineered MSCs and monitoring the therapeutic effect of the cells simultaneously *in vitro* and *in vivo*.

## Supporting information

S1 Fig Characterization of transduced CAL62 cells, with double fusion (DF) proteins. (A) Scheme for the lentiviral reporter gene containing mCherry and Rluc driven by the CMV promoter. (B) Fluc activity and quantitative analysis of stably transduced CAL62 cells and relationship to cell number. (C) Transduced CAL62 cells are strongly positive for mCherry by fluorescence microscopy (D) RT-PCR analysis of Rluc gene. (E) Detection of the Rluc protein by western blot.
(TIF)

S2 Fig Fluc activity after induction and withdrawal of DOX in MSC-Tet-TK-Fluc cells. (A) Fluc activity in the presence of various concentrations of DOX after 24, 48, and 72 h measured using BLI imaging; quantitation of Fluc activity is expressed as fold change. (B) Time course of Fluc activity following withdrawal of DOX 24 h after initial DOX induction. Remaining Fluc activity was detected 24 and 48 h later by BLI imaging and quantitation of the Fluc activity is expressed as fold change.
(TIF)

S3 Fig *In vitro* analysis of MSC-Tet-TK/Fluc and MSC-TK/Fluc bystander killing of CAL62 tumor cells after treatment with 8 μM GCV for 48 h. Image of (A) Untreated MSC-Tet-TK/Fluc and CAL62/Rluc cells. (B) DOX treated MSC-Tet-TK/Fluc and CAL62/Rluc cells. (C) MSC-TK/Fluc and CAL62/Rluc cells. All images were taken at 20x magnification using fluorescence microscopy.
(TIF)

## References

[1] Strachan T, Read AP. Gene therapy and other molecular genetic-based therapeutic approaches. 1999.

[2] Amer MH. Gene therapy for cancer: present status and future perspective. Molecular and cellular therapies. 2014;2(1):1. 10.1186/2052-8426-2-27 PMC4452068 26056594

[3] Weichselbaum RR, Kufe D. Gene therapy of cancer. The Lancet. 1997;349:S10–S2. 10.1016/s0140-6736(97)90013-1 9164440

[4] Goverdhana S, Puntel M, Xiong W, Zirger J, Barcia C, Curtin JF, et al Regulatable gene expression systems for gene therapy applications: progress and future challenges. Molecular Therapy. 2005;12(2):189–211. doi: 10.1016/j.ymthe.2005.03.022 15946903 10.1016/j.ymthe.2005.03.022 PMC2676204

[5] Kis Z, Pereira HSA, Homma T, Pedrigi RM, Krams R. Mammalian synthetic biology: emerging medical applications. Journal of The Royal Society Interface. 2015;12(106):20141000. 10.1098/rsif.2014.1000 PMC4424663 25808341

[6] Bonini C, Bondanza A, Perna SK, Kaneko S, Traversari C, Ciceri F, et al The suicide gene therapy challenge: how to improve a successful gene therapy approach. Mol Ther. 2007;15(7):1248–52. doi: 10.1038/sj.mt.6300190 17505474 10.1038/sj.mt.6300190

[7] Leng L, Wang Y, He N, Wang D, Zhao Q, Feng G, et al Molecular imaging for assessment of mesenchymal stem cells mediated breast cancer therapy. Biomaterials. 2014;35(19):5162–70. doi: 10.1016/j.biomaterials.2014.03.014 24685267 10.1016/j.biomaterials.2014.03.014 PMC4414495

[8] Gebremedhin S, Singh A, Koons S, Bernt W, Konopka K, Duzgunes N. Gene delivery to carcinoma cells via novel non-viral vectors: Nanoparticle tracking analysis and suicide gene therapy. Eur J Pharm Sci. 2014;60:72–9. doi: 10.1016/j.ejps.2014.03.003 24751674 10.1016/j.ejps.2014.03.003

[9] Matuskova M, Kozovska Z, Toro L, Durinikova E, Tyciakova S, Cierna Z, et al Combined enzyme/prodrug treatment by genetically engineered AT-MSC exerts synergy and inhibits growth of MDA-MB-231 induced lung metastases. J Exp Clin Cancer Res. 2015;34(1):1. 25884597 10.1186/s13046-015-0149-2 PMC4431639

[10] Kucerova L, Matuskova M, Pastorakova A, Tyciakova S, Jakubikova J, Bohovic R, et al Cytosine deaminase expressing human mesenchymal stem cells mediated tumour regression in melanoma bearing mice. The journal of gene medicine. 2008;10(10):1071–82. doi: 10.1002/jgm.1239 18671316 10.1002/jgm.1239

[11] Cavarretta IT, Altanerova V, Matuskova M, Kucerova L, Culig Z, Altaner C. Adipose tissue–derived mesenchymal stem cells expressing prodrug-converting enzyme inhibit human prostate tumor growth. Mol Ther. 2010;18(1):223–31. doi: 10.1038/mt.2009.237 19844197 10.1038/mt.2009.237 PMC2839205

[12] de Melo SM, Bittencourt S, Ferrazoli EG, da Silva CS, da Cunha FF, da Silva FH, et al The anti-tumor effects of adipose tissue mesenchymal stem cell transduced with HSV-Tk gene on u-87-driven brain tumor. PLoS One. 2015;10(6):e0128922 doi: 10.1371/journal.pone.0128922 26067671 10.1371/journal.pone.0128922 PMC4467037

[13] Barese CN, Krouse AE, Metzger ME, King CA, Traversari C, Marini FC, et al Thymidine kinase suicide gene-mediated ganciclovir ablation of autologous gene-modified rhesus hematopoiesis. Mol Ther. 2012;20(10):1932–43. doi: 10.1038/mt.2012.166 22910293 10.1038/mt.2012.166 PMC3464648

[14] Kucerova L, Matuskova M, Hlubinova K, Altanerova V, Altaner C. Tumor cell behaviour modulation by mesenchymal stromal cells. Mol Cancer. 2010;9(1):1. 20509882 10.1186/1476-4598-9-129 PMC2890609

[15] Yong RL, Shinojima N, Fueyo J, Gumin J, Vecil GG, Marini FC, et al Human bone marrow–derived mesenchymal stem cells for intravascular delivery of oncolytic adenovirus Δ24-RGD to human gliomas. Cancer Res. 2009;69(23):8932–40. doi: 10.1158/0008-5472.CAN-08-3873 19920199 10.1158/0008-5472.CAN-08-3873 PMC2789204

[16] Nakamizo A, Marini F, Amano T, Khan A, Studeny M, Gumin J, et al Human bone marrow–derived mesenchymal stem cells in the treatment of gliomas. Cancer Res. 2005;65(8):3307–18. doi: 10.1158/0008-5472.CAN-04-1874 15833864 10.1158/0008-5472.CAN-04-1874

[17] Mader EK, Maeyama Y, Lin Y, Butler GW, Russell HM, Galanis E, et al Mesenchymal stem cell carriers protect oncolytic measles viruses from antibody neutralization in an orthotopic ovarian cancer therapy model. Clin Cancer Res. 2009;15(23):7246–55. doi: 10.1158/1078-0432.CCR-09-1292 19934299 10.1158/1078-0432.CCR-09-1292 PMC2787715

[18] Josiah DT, Zhu D, Dreher F, Olson J, McFadden G, Caldas H. Adipose-derived stem cells as therapeutic delivery vehicles of an oncolytic virus for glioblastoma. Mol Ther. 2010;18(2):377–85. doi: 10.1038/mt.2009.265 19904233 10.1038/mt.2009.265 PMC2839314

[19] Dwyer RM, Khan S, Barry FP, O'Brien T, Kerin MJ. Advances in mesenchymal stem cell-mediated gene therapy for cancer. Stem Cell Research & Therapy. 2010;1(3):1. 10.1186/scrt25 PMC2941117 20699014

[20] Fillat C, Carrio M, Cascante A, Sangro B. Suicide gene therapy mediated by the Herpes Simplex virus thymidine kinase gene/Ganciclovir system: fifteen years of application. Curr Gene Ther. 2003;3(1):13–26. 12553532 10.2174/1566523033347426

[21] Su H, Forbes A, Gambhir SS, Braun J. Quantitation of cell number by a positron emission tomography reporter gene strategy. Mol Imaging Biol. 2004;6(3):139–48. doi: 10.1016/j.mibio.2004.02.001 15193248 10.1016/j.mibio.2004.02.001

[22] Wang L, Wang Y, Li Z. Nanoparticle-based tumor theranostics with molecular imaging. Curr Pharm Biotechnol. 2013;14(7):683–92. 24372235 10.2174/1389201014666131226111248

[23] Pellegriti G, Frasca F, Regalbuto C, Squatrito S, Vigneri R. Worldwide increasing incidence of thyroid cancer: update on epidemiology and risk factors. Journal of cancer epidemiology. 2013;2013:965212 doi: 10.1155/2013/965212 23737785 10.1155/2013/965212 PMC3664492

[24] Patel KN, Shaha AR. Poorly differentiated and anaplastic thyroid cancer. Cancer Control. 2006;13(2):119–28. 16735986 10.1177/107327480601300206

[25] Gossen M, Bujard H. Tight control of gene expression in mammalian cells by tetracycline-responsive promoters. Proceedings of the National Academy of Sciences. 1992;89(12):5547–51. 10.1073/pnas.89.12.5547 PMC49329 1319065

[26] Gossen M, Freundlieb S, Bender G, Muller G, Hillen W, Bujard H. Transcriptional activation by tetracyclines in mammalian cells. Science. 1995;268(5218):1766–9. 7792603 10.1126/science.7792603

[27] Urlinger S, Baron U, Thellmann M, Hasan MT, Bujard H, Hillen W. Exploring the sequence space for tetracycline-dependent transcriptional activators: novel mutations yield expanded range and sensitivity. Proceedings of the National Academy of Sciences. 2000;97(14):7963–8. 10.1073/pnas.130192197 PMC16653 10859354

[28] Zhou X, Vink M, Klaver B, Berkhout B, Das A. Optimization of the Tet-On system for regulated gene expression through viral evolution. Gene Ther. 2006;13(19):1382–90. doi: 10.1038/sj.gt.3302780 16724096 10.1038/sj.gt.3302780

[29] Loew R, Heinz N, Hampf M, Bujard H, Gossen M. Improved Tet-responsive promoters with minimized background expression. BMC biotechnology. 2010;10(1):81. 21106052 10.1186/1472-6750-10-81 PMC3002914

[30] Sekar TV, Foygel K, Ilovich O, Paulmurugan R. Noninvasive theranostic imaging of HSV1-sr39TK-NTR/GCV-CB1954 dual-prodrug therapy in metastatic lung lesions of MDA-MB-231 triple negative breast cancer in mice. Theranostics. 2014;4(5):460–74. doi: 10.7150/thno.8077 24669276 10.7150/thno.8077 PMC3964441

[31] Nouri FS, Wang X, Hatefi A. Genetically engineered theranostic mesenchymal stem cells for the evaluation of the anticancer efficacy of enzyme/prodrug systems. J Control Release. 2015;200:179–87. doi: 10.1016/j.jconrel.2015.01.003 25575867 10.1016/j.jconrel.2015.01.003 PMC4758350

[32] Nakamura K, Ito Y, Kawano Y, Kurozumi K, Kobune M, Tsuda H, et al Antitumor effect of genetically engineered mesenchymal stem cells in a rat glioma model. Gene Ther. 2004;11(14):1155–64. doi: 10.1038/sj.gt.3302276 15141157 10.1038/sj.gt.3302276

[33] Ahn B-C. Sodium iodide symporter for nuclear molecular imaging and gene therapy: from bedside to bench and back. Theranostics. 2012;2(4):392–402. doi: 10.7150/thno.3722 22539935 10.7150/thno.3722 PMC3337731

[34] Kim JE, Kalimuthu S, Ahn B-C. In Vivo Cell Tracking with Bioluminescence Imaging. Nucl Med Mol Imag. 2015;49(1):3–10. 10.1007/s13139-014-0309-x PMC4354780 25774232

[35] Smallridge RC, Ain KB, Asa SL, Bible KC, Brierley JD, Burman KD, et al American Thyroid Association guidelines for management of patients with anaplastic thyroid cancer. Thyroid. 2012;22(11):1104–39. doi: 10.1089/thy.2012.0302 23130564 10.1089/thy.2012.0302

[36] Rodenhuis S, Bontenbal M, Beex LV, Wagstaff J, Richel DJ, Nooij MA, et al High-dose chemotherapy with hematopoietic stem-cell rescue for high-risk breast cancer. N Engl J Med. 2003;349(1):7–16. doi: 10.1056/NEJMoa022794 12840087 10.1056/NEJMoa022794

[37] Kim KB, Eton O, East MJ, Hodges C, Papadopoulos NE, Grimm EA, et al Pilot study of high‐dose, concurrent biochemotherapy for advanced melanoma. Cancer. 2004;101(3):596–603. doi: 10.1002/cncr.20403 15274073 10.1002/cncr.20403

[38] Zeng Z, Li Z, Luo S, Hu W. Retrovirus-mediated tk gene therapy of implanted human breast cancer in nude mice under the regulation of Tet-On. Cancer Gene Ther. 2006;13(3):290–7. doi: 10.1038/sj.cgt.7700889 16110312 10.1038/sj.cgt.7700889

[39] Schmeisser F, Donohue M, Weir JP. Tetracycline-regulated gene expression in replication-incompetent herpes simplex virus vectors. Hum Gene Ther. 2002;13(18):2113–24. doi: 10.1089/104303402320987815 12542843 10.1089/104303402320987815

[40] Koponen J, Kankkonen H, Kannasto J, Wirth T, Hillen W, Bujard H, et al Doxycycline-regulated lentiviral vector system with a novel reverse transactivator rtTA2S-M2 shows a tight control of gene expression in vitro and in vivo. Gene Ther. 2003;10(6):459–66. doi: 10.1038/sj.gt.3301889 12621450 10.1038/sj.gt.3301889

[41] Mizuguchi H, Xu Z-L, Sakurai F, Mayumi T, Hayakawa T. Tight positive regulation of transgene expression by a single adenovirus vector containing the rtTA and tTS expression cassettes in separate genome regions. Hum Gene Ther. 2003;14(13):1265–77. doi: 10.1089/104303403767740803 12952598 10.1089/104303403767740803

[42] Klopp AH, Gupta A, Spaeth E, Andreeff M, Marini F. Concise review: dissecting a discrepancy in the literature: do mesenchymal stem cells support or suppress tumor growth? Stem cells. 2011;29(1):11–9. doi: 10.1002/stem.559 21280155 10.1002/stem.559 PMC3059412

[43] Torsvik A, Bjerkvig R. Mesenchymal stem cell signaling in cancer progression. Cancer Treat Rev. 2013;39(2):180–8. doi: 10.1016/j.ctrv.2012.03.005 22494966 10.1016/j.ctrv.2012.03.005

[44] Amano S, Li S, Gu C, Gao Y, Koizumi S, Yamamoto S, et al Use of genetically engineered bone marrow-derived mesenchymal stem cells for glioma gene therapy. Int J Oncol. 2009;35(6):1265 19885548 10.3892/ijo_00000443

[45] Vilalta M, Degano I, Bago J, Aguilar E, Gambhir S, Rubio N, et al Human adipose tissue-derived mesenchymal stromal cells as vehicles for tumor bystander effect: a model based on bioluminescence imaging. Gene Ther. 2009;16(4):547–57. doi: 10.1038/gt.2008.176 19092860 10.1038/gt.2008.176

[46] Zhang T-Y, Huang B, Wu H-B, Wu J-H, Li L-M, Li Y-X, et al Synergistic effects of co-administration of suicide gene expressing mesenchymal stem cells and prodrug-encapsulated liposome on aggressive lung melanoma metastases in mice. J Control Release. 2015;209:260–71. doi: 10.1016/j.jconrel.2015.05.007 25966361 10.1016/j.jconrel.2015.05.007

[47] Latta-Mahieu M, Rolland M, Caillet C, Wang M, Kennel P, Mahfouz I, et al Gene transfer of a chimeric trans-activator is immunogenic and results in short-lived transgene expression. Hum Gene Ther. 2002;13(13):1611–20. doi: 10.1089/10430340260201707 12228016 10.1089/10430340260201707

[48] Han Y, Chang QA, Virag T, West NC, George D, Castro MG, et al Lack of humoral immune response to the tetracycline (Tet) activator in rats injected intracranially with Tet-off rAAV vectors. Gene Ther. 2010;17(5):616–25. doi: 10.1038/gt.2010.6 20164859 10.1038/gt.2010.6 PMC2869394

[49] Hoyng SA, Gnavi S, de Winter F, Eggers R, Ozawa T, Zaldumbide A, et al Developing a potentially immunologically inert tetracycline-regulatable viral vector for gene therapy in the peripheral nerve. Gene Ther. 2014;21(6):549–57. doi: 10.1038/gt.2014.22 24694534 10.1038/gt.2014.22

