# Supplementary figures and images for: Genetically engineered suicide gene in mesenchymal stem cells using a Tet-On system for anaplastic thyroid cancer
Source: PLoS One. 2017 Jul 20;12(7):e0181318. doi: 10.1371/journal.pone.0181318 (PMC5519161; doi:10.1371/journal.pone.0181318)

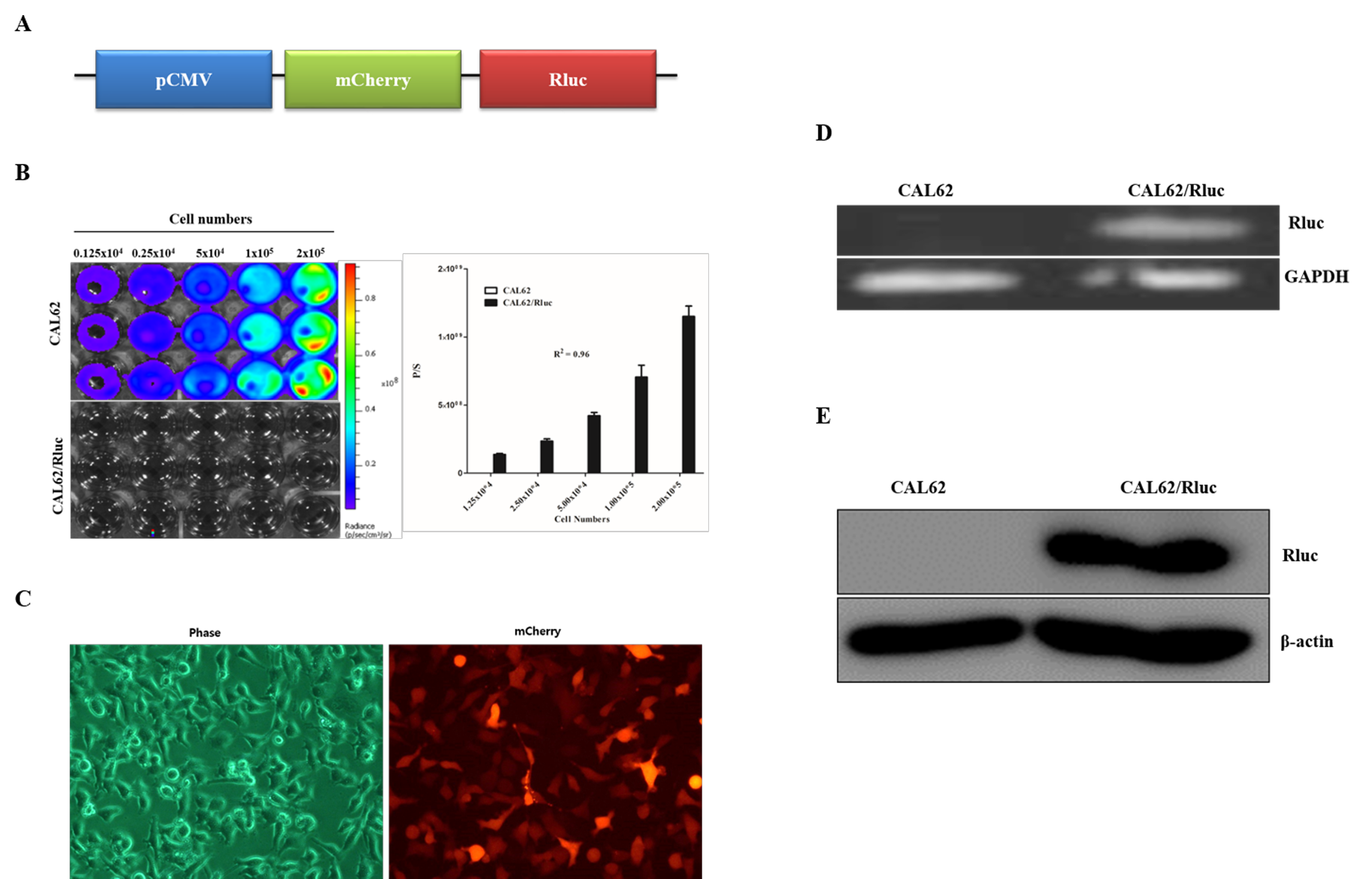

Supplement: S1 Fig — (A) Scheme for the lentiviral reporter gene containing mCherry and Rluc driven by the CMV promoter. (B) Fluc activity and quantitative analysis of stably transduced CAL62 cells and relationship to cell number. (C) Transduced CAL62 cells are strongly positive for mCherry by fluorescence microscopy (D) RT-PCR analysis of Rluc gene. (E) Detection of the Rluc protein by western blot. (TIF) [file pone.0181318.s001.tif]

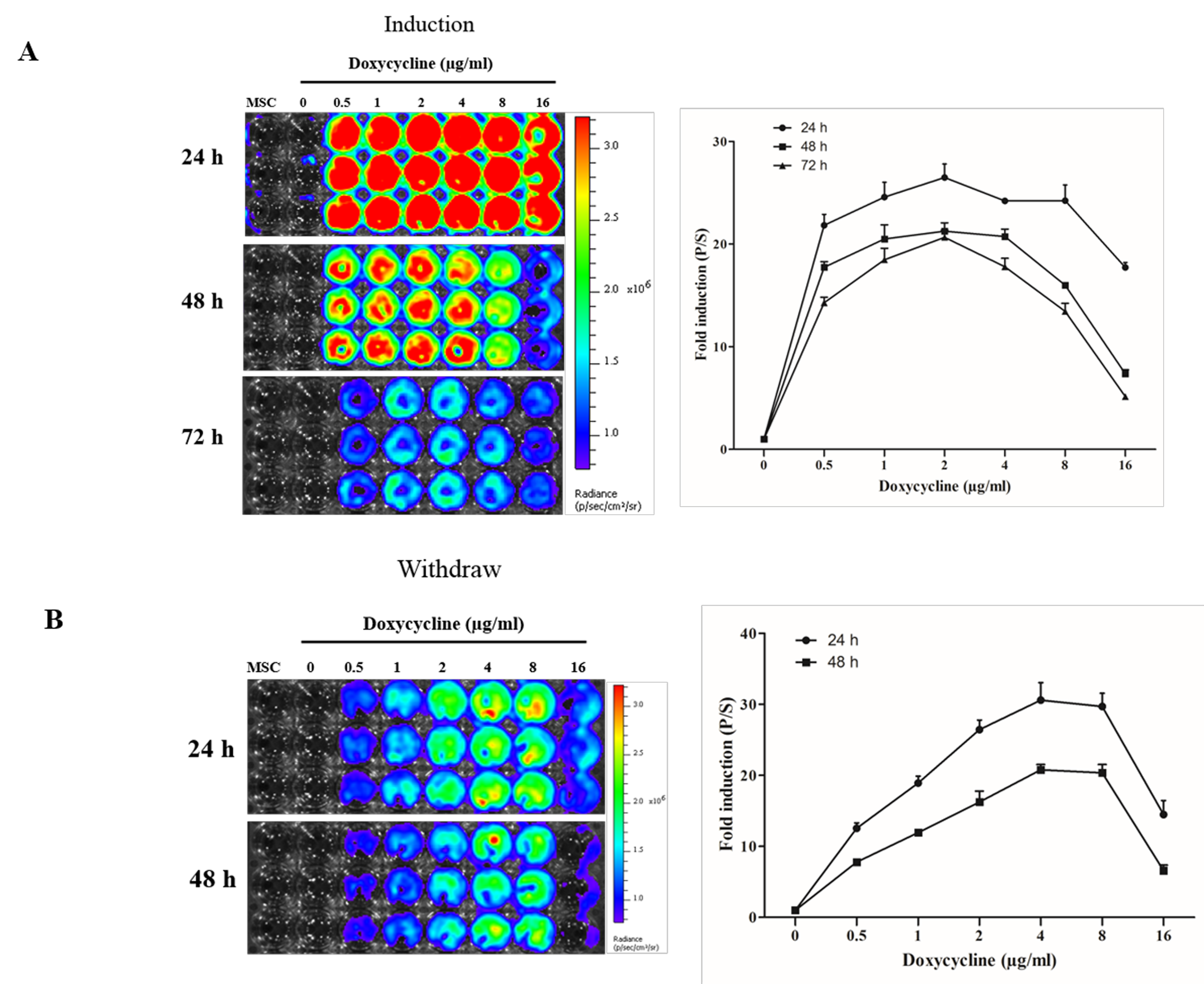

Supplement: S2 Fig — (A) Fluc activity in the presence of various concentrations of DOX after 24, 48, and 72 h measured using BLI imaging; quantitation of Fluc activity is expressed as fold change. (B) Time course of Fluc activity following withdrawal of DOX 24 h after initial DOX induction. Remaining Fluc activity was detected 24 and 48 h later by BLI imaging and quantitation of the Fluc activity is expressed as fold change. (TIF) [file pone.0181318.s002.tif]

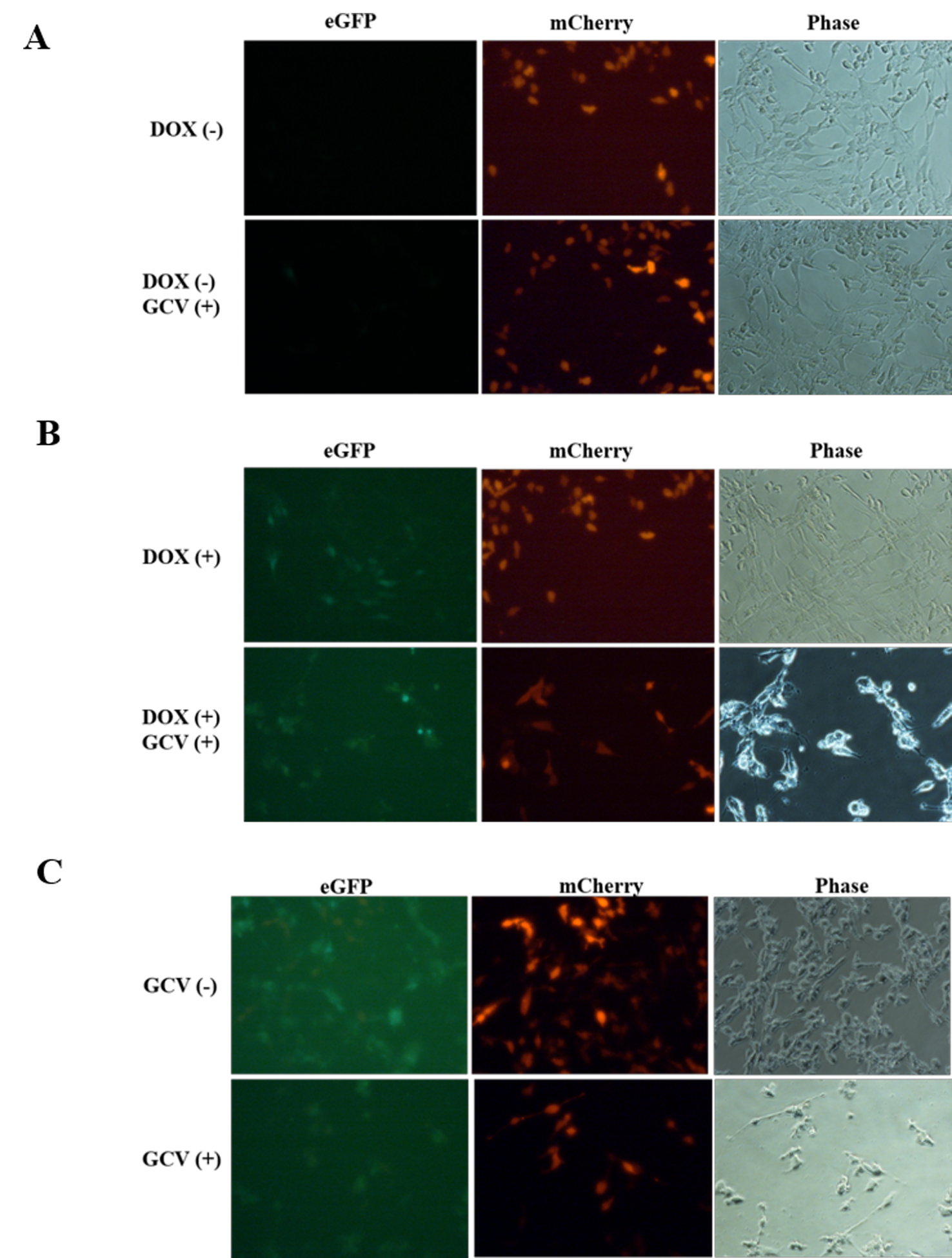

Supplement: S3 Fig — Image of (A) Untreated MSC-Tet-TK/Fluc and CAL62/Rluc cells. (B) DOX treated MSC-Tet-TK/Fluc and CAL62/Rluc cells. (C) MSC-TK/Fluc and CAL62/Rluc cells. All images were taken at 20x magnification using fluorescence microscopy. (TIF) [file pone.0181318.s003.tif]
